# Experimental and Microscopical Researches in Traumatic Injuries of the Liver and Hepatic Abscess

**Published:** 1882-10

**Authors:** W. S. Elkin

**Affiliations:** Atlanta, Ga.


					﻿ATLANTA
Medical Register.
Vol. II.]	OCTOBER, 1882.	[No. 1
Original.
EXPERIMENTAL AND MICROSCOPICAL RISE ARCH-
ES IN TRAUMATIC INJURIES OF TII (•
LIVER AND HEPATIC ABSCESS.
By W. 3. ELKIN, M.D., Atlanta, Ga.
Read before the Atlanta Medical Society.
I	have not only selected this subject to familiarize my-
self with these important troubles, but I shall, at the san
time, strive to throw light upon some paramount facts
that remain as yet a question of dispute.
By a series of careful experiments I think I am able
to prove that gun shot wounds of the liver, not involv-
ing its large vessels, are not fatal, and that they will
heal without the development of any serious constitu-
tional symptoms. Furthermore, I will endeavor to show
that we can detect in the pus from liver abscesses liver
cells—which fact will be an invaluable diagnostic point
in these diseases.
My experiments were done in the Pathological Labora-
tory of the University of Pennsylvania, and my results
were all verified by Dr. Formad, the Microscopist to that
institution.
Part I.
Traumatic Injuries of the Liver.—The animals selected
for experimental purposes were dogs, whose livers are
comparatively large, and, by a careful process of palpa-
tion and percussion, can be readily outlined, and hence
may be lacerated, punctured, shot through, or injected
with some cauterizing agent, without fatal results. In
each experiment the animal was profoundly etherized,
placed on the left side, and, after the exact position of the
liver uad been mapped out, this organ was either shot
through or had injected into its structure tincture of can-
tharides.
Gun-shot wounds were inflicted on the livers of large
dogs, while irritants were resorted to in the cases of small
dogs.
In shooting the animals, care was taken not to wound
any of the larger blood vessels of the liver, or injure any
of the surrounding organs. To accomplish this, the ball
was made to enter the body of the animal between the
seventh and eighth or eighth and ninth ribs, and range
so as to penetrate the liver and lodge in the muscles of the
back, to the right of the spinal column. The size of the
cartridge ball was No. 22, long.
Injections were made in the liver, usually, between the
seventh and eighth ribs. The hypodermic syringe used
for this purpose had, instead of the ordinary short, deli-
cate needle, a large, stout one, two and a half inches long,
which could be made to penetrate deep into the liver
structure. The irritant used was tincture of cantharides,
U. S. P.
The animals were kept alive from ten to twenty-five
days. Immediately after the death of the dog the liver
was removed and put in dilute, and, after twenty-four
hours, into stronger alcohol.
After the liver had become sufficiently hard the sec-
tions were cut and mounted in damar varnish, and a care-
ful examination was made with a microscope of a magni-
fying power of from 100 to 1,000 diameters.
The following table shows the experiments and the
Tesults :
’	j
O O O © O O O r,
.......................... Experiment.
Q	CO tO	—
— — j—i j—i — —	r"*l
» ,	S’<■	<	I	Or«a?.	,
© © © n a> ©	©	Injured.
"* © r* r« 7* 7>	r»	|
c Ci " cn cn	oo	i	Days._
Q M X Q OC Q QI ,
~ ~ ~ ~ ~	-	2	Gunshot	or
<—». c** <■■*• c*	c*-	<-■*■	Matter	In-
“	* ~	“	jected.
«, -b ■?	Size of Ball
2	•	21?	2	2	or quanti
tctc tc'	__tyjn jected.
c © o c o o' o' Animal.
TJ J5 0Q 'JK 7Q 7Q 0q
CO Q f > Q
*-»>2><E. 2 ^'c^.3 ►—< 2
' © c—‘ — —» © O S © CX
2	2 -i 2 — n *’■^2
—1 —.	• r— Z —
■ © X5 —- x x — © « zr.
I- * 2 2 3 ® E £• 2 w
E©—©
r* * CL	□	•—'
C. "	<-»►—< —	<7**
M8=.®S-°“
2	5^2,2 “B	-z
© ©	x	©	©	»=	-	“«
~ E=	-	©	©	^3	°
J	.7 i 3 -	©
3	©	> ST.	jf	©
°	~	~	c
sr.	*	5
s	=	~
‘i	~©	©^|
Experiment No 1. Dog,	weight, 11 pounds. On No-
vember 11th, injected into the liver f3j tincture canthar-
ides, and killed the animal November 18th.
Upon removal, the organ had a highly congested appear-
ance with adhesive inflammation extending tosurrounding
parts. Capsule quite adherent, dark appearance, very
friable, finger sinking deeply into the structure when
pressed upon. Blood exuded when a section was made,
and the gall bladder was found partly filled. After a care-
ful microscopical examination, the liver was placed first
in dilute and then in stronger alcohol.
Microscope showed strong intestinal inflammation with
catarrhal hepatitis. Liver cells greatly enlarged with
nuclei plainly visible, lobule distorted, also bile infarct.
Cells in all three cases about alike—pressed apart and
widely separated. Connective tissue increased around
inter and intra-lobular vessels, and the stroma of connec-
tive tissue was infiltrated with embryonic and stellate
cells, scattered between liver cells. Blood vessels were
distended with blood, interlobular connective tissue in-
creased, and infiltrated with cells. No abscess anywhere,
only a state of high congestion with above changes.
Experiment No. 2. Small dog, weight, 12 pounds. In-
jected tincture of cantharides f3j into the liver on Novem-
ber 15th and repeated the experiment with the same
amount of tincture of cantharides on November 25th,
killed the dog December 11th.
Removed the liver immediately after death. Macro-
scopical appearance ; organ gave hobnailed appearance,
quite anaemic, rather tough on cutting—very little blood
oozing. Adhesive inflammation around the organ, cap-
sule adherent in many places, did not pit on pressure.
Gall bladder had little bile in it. Hardened the organ in
alcohol and sections made, which showed under miscro-
scope lobules somewhat distorted, cells pressed together,
and elongated liver cells, jaundiced; granular nucleus dis-
tinguishable between the cells and between the lobules—
more especially the latter. There was an increase of con-
nective tissue with cells; cells alike in all three zones.
Hepatic veins surrounded by an increase of spindle shaped
cells, no blood in blood vessels, no blood infarcts, re-
semble very much cirrhosed liver.
Experiment No. 3. A large white dog, shot through
the liver on December 1st and killed December 16th.
The liver was very dark, highly congested and of a
nutmeg appearance. On section, very dark blood oozed
out of the veins. Gall bladder distended with bile and
strong adhesions between the liver and peritoneum on one
hand, and the peritoneum and kidney on the other. Ad-
hesions also to the diaphragm, and to the small intestines,
so that these organs could not be separated from each other
without tearing. There was seen a large partly healed
scar in that portion which was attached to the kidney.
The cicatrix was deeply stained with bile—the staining
extending to the capsule of the kidney and to the adhesions
between the organs. In a portion of the scar there was
a triangular infarct measuring about one-third of an inch
at the base—but no softening around the part. The cap-
sule of the liver was also the seat of several superficial
scars; and at the lower portion of the left lobe the whole
edge of the liver was replaced by a large depressed scar.
A microscopical examination of this scar revealed the fol-
lowing : There was seen a large irregular band of con-
nective tissue plainly marking that portion of the scar
from which the section had been made. The scar tissue
contained besides connective tissue cells, granular matter
and some broken down liver cells which were very much
elongated.
Neither abscess nor cheesy substance could be found
any where. The right kidney (which has been described
as being adherent to the liver by means of the perito-
neum and inflammatory adhesions) showed a very much
thickened and congested capsule, and a large hemorrhagic
infarct in corresponding portion of the kidney. The in-
farcted place appeared as a hardened yellow lump about
one-half an inch in diameter.
On the second and third days after the animal was shot he
was in a very critical condition, and I thought at one time
he would surely die, but on the fourth day he was some-
what better and from this time on steadily improved to
recovery, and on the day he was killed, which was the
fifteenth day after the injury, he was entirely well. On the
fourth day after the injury I led the dog out of the pen, in
order to examine his wound, and while out he urinated.
During thi3 act of micturition I succeeded, by placing a
basin beneath the animal, in catching several ounces of his
urine. It was dark and highly colored. Placing a drop
of it on a plate and adding a drop of nitric acid, a char-
acteristic play of colors was seen, showing the presence of
bile. Under the microscope epithelial tube casts and
cholesterin plates were revealed. Albumen was also found
in the urine.
Experiment No. 4. On December Gth injected f3j of
tincture of cantharides into the liver of a small black dog,
and killed the animal December 29th.
Autopsy made immediately after death. A few in-
flammatory adhesions, and capsule was closely adherent.
Macroscopical appearance of liver was congested and on
section dark blood oozed from veins. Gall bladder was
partially filled with bile. Near the centre of the left lobe
where the cantharides was injected was a circumscribed
abscess about half of an inch in diameter and from a quar-
ter to half an inch in depth. It was enclosed in a capsule
slightly projecting above the surface of the liver. On
cutting through the abscess, the contents were found to be
dry and hard, and undergoing, as it wrere, a process of
natural healing.
A microscopical examination of a section of this cir-
cumscribed area and surrounding tissue showed the fol-
lowing : In the abscess proper we found blood corpuscles,
pus cells, and portions of broken down liver structure-
Surrounding this tissue area we found a great quantity of
connective tissue, containing granular matter and a few
degenerated liver cells without the stroma connective.
We also observed normal liver structure. This band of
scar tissue is conclusive evidence that the abscess had
been, and was still probably, undergoing a process of
healthy healing, or cicatrization. The dog did not suffer
any contstitutional trouble at any time, from this injury.
He lived three weeks, and when killed was in apparent
good health.
Experiment No. 5. A large black dog very stout, shot
through the liver December 10th and killed December
15th.
Autopsy showed a highly congested liver, with ad-
hesions to diaphragm and peritoneum. The liver was soft
and pitted on pressure and very dark red blood exuded on
section. The capsule of the liver adherent very closely.
On the lower border of the right lobe of the liver was.
seen a superficial scar half an inch in diameter, but only
a few linesin thickness. It seemed only to extend through
the thickness of the capsule. The ball merely grazed this
portion of the liver, passing on and lodging in the muscles
of the back, where it had become encysted. The wound
healed very quickly, as it was only five days since it w’as
inflicted and at the autopsy the external appearance was
that of a perfect cacatrix. The microscope showed scar
tissue. The wound inflicted in the parietes by the ball
was not completely healed but was undergoing a state of
healthy granulation. The inflammatory adhesions were
due, in my mind, to the inflammation of the surrounding
structure caused by the passage of the ball through the
body of the animal. The dog did not suffer any constitu-
tional symptoms. Other organs were examined, but found
normal.
Experiment No. 6.—Large black bitch, shot through
the liver and also injected into this organ fSj of tinc-
ture of cantharides. The experiment was made December
11th; killed the dog December 27th; autopsy made im-
mediately after death. Both thoracic and abdominal cav-
ities contained fluid and coagulated blood. No adhesions
about the liver; in color the organ w’as normal.
There were two wounds in the left lobe of the liver—
one in the middle, which proved to be the first injury, and
the other in the left lower edge, which was the last in-
jury, and the one that caused the animal’s death. On the
right upper part of the left lobe there was a peripheral
node of cheesy matter, infiltrating the capsule and liver
structure beneath, and being about the size of a hazel-nut.
A small hemorrhagic cyst, about an eighth to a quarter of
an inch in diameter, was seen in the upper portion of the
left lobe, near the diaphragm.
Wound No. 1 was perfectly healed, and showed a scar.
No trace of the ball; it only struck the organ at tlfis place
without entering into the structure of the liver. Under a
microscope the scar showed cicatricial tissue, intermin-
gled with elements of organized blood clot and liver cells*
Wound No. 2 was made two weeks after the first wound,
and was a perfectly fresh one; the edges were ragged
The ball went through the liver and injured the abdomi-
nal aorta. There was great hemorrhage, causing the
death of the animal. The ball probably passed out of
the body, as it could not be found. Lesion No. 3, or the
cheesy node that appeared on the left lobe of the liver,
was diffused infiltration of cells in the connective tissue
of the liver without producing any perceptible loss of
substance. A microscopical preparation of this white
nodule showed an infiltration of young connective tissue
cells, partly undergoing a higher organization and partly
fatty degeneration, and stained with biliary coloring mat-
ter, while in some places it showed clotted blood, inter-
mingled with fibrine. Around this there was a more or
less dense fibrinous capsule, surrounded by elongated liver
cells, which had acquired this shape from pressure; and
the blood vessels showed evidence of a decided hypersemia
all around the lesion. The gall bladder was filled, and
there was some slight adipose tissue between it and the
liver substance. The blood vessels of its mucous mem-
brane were congested. Kidneys were of normal appear-
ance, except cortical portion, which was more or less
congested. Kidneys showed a few white nodules just
beneath the capsule, and a cyst filled with clear liquid
was situated on the periphery. The other organs were
normal.
Experiment No. 7.—Small dog; injected into his liver,
on December 13th, f3j of tincture of cantharides; killed,
December 22d. Organ not congested, but softer and more
flabby than normal; color, grayish-brown hue, and but
little oozing on cutting; quite friable. The gall bladder
and the liver pitted on pressure; capsule stripped off
easily; liver cells stained with bile; cloudy and enlarged
nucleus observed ; biliary matter and granular depos-
it; often wide interspaces between the lobules; bile
infarct and gall duct surrounded by embryonic tissue. No
increase of interlobular connective tissue. Liver cells more
widely separated and granulated near hepatic vein, grad-
ually decreasing towards portal vein. Embryonic tis-
sue around portal vein and hepatic artery. No inflam-
matory adhesions, and other organs normal.
Conclusions.—The above experiments show : 1st. That
the injuries of the liver heal in precisely the same man-
ner as external wounds of the body heal. 2d. Hepatic
abscesses are not produced from gun-shot wounds of the
liver. 3d. Gun-shot wounds of the liver do not produce
any grave constitutional symptoms, and unless other vital
parts are wounded, do not terminate fatally. 4th. The
earliest time for healing of injuries of the capsule is five
days, and of the true liver structure, fifteen days. 5th.
The injection of simple irritating fluids into the substance
of the liver produces similar results to those noted above.
Part II.
Observations Upon a Case of Hepatic Abscess.—Unfortu-
nately my observation is limited to one case, but after
carefully investigating it, I am able to offer some conclu-
sive evidence concerning the microscopical diagnosis of
this lesion.
Case.—Theodore Wolberg, German, 37 years of age*
admitted to the Philadelphia Hospital October 15th, 1881.
He was a resident of the United States thirteen years;
business blacksmithing. Healthy parents, and he was
never sick excepting with chills and fever five years be-
fore. He worked most of his time in Philadelphia, at
carriage blacksmithing. Five and a half months ago he
was taken sick with pain in head and some fever, consti-
pated for four days, receiving relief from a dose of physic
and when, after resting two days, went to work at a very
heavy job of welding ties for a wagon and tiring the
wheels.
U;x>n finishing this job he felt a dull pain beginning
two inches to the right, and above the umbilicus and ex-
tending around to the right side between the eleventh and
twelfth ribs. Was in bed from Saturday until Monday
morning. Was admitted to the St. Mary’s Hospital, May,
10th, 1881, at which time he had been suffering for forty-
eight hours from retention of urine, which had to be with-
drawn with a catheter. After three weeks treatment in St.
Mary’s Hospital he was pronounced convalscent, and went
home to engage in some light employment. At the ex-
piration of one week he came to the Philadelphia Hos-
pital remaining seven weeks, when he improved in health
and was discharged. For three weeks he was again em-
ployed at some light work, when, on return of the same
pain in right hypochondriac region, he was admitted to
the Pennsylvania Hospital where he remained eight weeks.
Feeling better he took his discharge, and engaged in
light work for three weeks when, the pain returning,
he came back to the Philadelphia Hospital, October 15th,
1881. On admission his face was pale and jaundiced, and
he complained of great pain in his right side. Upon ex-
amination there was found tenderness in front and bulging
of the eleventh and twelfth ribs, with signs of fluctuation.
The pain continuing, the abscess was tapped and twenty
and a half fluid ounces of pus, of a light chocolate color,
was drawn off. This was done October 17th when he ob-
tained great relief. His temperature was ranging from 98°
F. in morning, to 101° in evening. His appetite has been
generally good. No diarrhoea, but on the contrary was
constipated. On the 24th of October, temperature was
99 1-5° F. Abscess bulging higher and pressing out tenth
and eleventh ribs, and showing more marked signs of
fluctuation. He felt pain in front, as well as behind. On
October 26th at 10 o’clock a. m. eighty-two fluid ounces of
pus was withdrawn, making in all one hundred and four
ounces. On October 29th the temperature was 99° to 100°
F. He complained of pain in the right hypochondriac
region, but his appetite was good and he was perfectly
comfortable. November 7th at 2:30 o’clock p. m. fifty fluid
ounces of pus was extracted niaking in all to that date
one hundred and fifty-four ounces. After aspiration felt
much more comfortable. An incision, so as to open the ab-
scess from without inwards, was made on the 10th. On the
evenings of the 15th and 16th and at 5 and 6 o’clock p.m. the
temperature was respectively 103 and 103 3-5°, but dropped
as usual in the morning. On November 19th the aspirator
was used through the incision already made and sixty-six
fluid ounces of chocolate colored pus was extracted, leaving
a considerable amount in the abscess. Temperature in the
evening 102° to 103°, falling in the morning to 99^. No-
vember 21st, twenty-eight fluid ounces of pus was with-
drawn—temperature 101°. He coughed considerable and
there was pain about the umbilicus. He lost flesh al-
though well fed. Appetite was generally good. Abscess
was syringed out morning and evening with a carbolized
solution of luke-warm water; and also with a solution of
iodoform. Glycerine and water was injected. Patient
continued to grow’ weaker, and on the 6th of December at
2:30 o’clock p. m. he died.
At post mortem examination liver was found to con-
tain multiple abscesses in right lobe. I should have men-
tioned before that the pus aspirated from the liver ab-
scess w’as repeatedly examined and, contrary to the ex-
perience of others, liver cells were invariably discovered.
I have not the slightest doubt that liver cells are found in
true liver abscesses, but that they are overlooked for
reasons that I will mention immediately. I had a large
number of specimens of pus taken from the above case,
at different times, and in my earliest examinations of this
pus I also failed to discover liver cells. In my later ex-
aminations, however, I saw liver cells after prolonged and
patient search. I discovered that the liver cells were ex-
tremely pale, and so faintly visible that I was not sur-
prised that I did not detect them at my previous examina-
tions. With a high magnifying power I noticed semi-
translucent bodies, frequently containing fat vesicles, and
sometimes presenting a milky, faintly granular appear-
ance. Occasionally a group of these translucent bodies
were seen together. In no case could I discover these if
I took a large drop of pus, so that everything was
crow’ded by leucocytes and granular debris. In minute
quantities, how’ever, diluted with a drop of distilled water,
the faint irregular bodies referred to, became distinct
enough to admit of observation. Upon careful study
these bodies proved to be liver cells, which had lost their
coloring matter from prolonged maceration in the serum.
I have convinced myself of the above, by scraping some
pus from the wall of the abscess of the liver, obtained
post mortem, from this case, and here I could distinctly
trace out the gradual transition between perfect liver
cells, and the faint, macerated-looking bodies which are
found in the contents of the abscess, and which are direct
derivations from the true liver cells. I regard the occurence
of fat drops in these bodies as a corrobative proof, as it
is impossible for any other detached cell to contain fat
drops of this character.
I should add yet, that I subsequently discovered such
pale macerated liver cells, even in specimens of pus
that had been standing for several weeks, and which in
former, less careful examinations, I had failed to notice.
Besides liver cells, are frequently found colestrin plates,
and I always saw multitudes of compound granular cells
and bacteria accompanied by molecular debris.
For the sake of completeness I will quote here some of
the authorities on this subject:
The first authority that claims to have observed cells
in pus from hepatic abscesses appears to be Samuel Fen-
wick, M.D., of London. He dwells at length upon several
cases in the London Lancet of November 17t.h, 1877,
the substance of which I will give below.
Fenwick states that it is a known fact to all that by
liquifying by means of soda the sputa of a patient affected
with phthisis, you can detect portions of the fibrous tissue of
the lungs; but you are unable to apply the same solvent to
the pus discharged from an hepatic abscess, because any
of the liver structure that may be present is readily dis-
solved by this process. You must, therefore, vary your
mode of procedure; and to explain how this can be
done, and also to point out what advantage may be gained
from this method of diagnosis, Fenwick reports several
cases and the results of his examinations. In case first,
which was diagnosed a liver abscess, some of the pus was
shaken up with distilled water and thrown into a conical
glass. In a few seconds, numerous fragments subsided,
which presented the appearance of liver mixed with
shreds of fibrous tissue. Examined microscopically, it
was quite evident the fragments were formed of hepatic
structure infiltrated with pus-cells, whilst the fibrous ma-
terial was similar to what is ordinarily found around old
abscesses of the liver. The pus was examined on several
occasions, and always with the same results. When you
have a large opening, and the ulceration, as in this in-
stance, is going on rapidly, the simple shaking up of the
pus with distilled water, and allowing the heavier parti-
cles to subside, will be sufficient to insure the detection of
liver structure. Dr. Fenwick reports another case in
which the symptoms of hepatic abscess were more obscure.
In this case the needle of an aspirator was passed into the
tumor, and withdrew six ounces of blood-stained pus. Some
of this pus was shaken up with water, but no particles of
liver could be detected. The remainder of the pus was,
therefore, digested for a few minutes along with distilled
water, and placed in a conical glass. Sediment subsided,
which, when examined microscopically, was proved to
contain a considerable number of minute fragments of
hepatic tissue. The patient rapidly sank, and on post-
mortem examination a large abscess was found in the
right lobe of the liver.
Here, you observe, the use of the microscope cleared
up all doubts as to the nature of the disease; but on ac-
count of the small size of the needle employed, the frag-
ments of the liver were so minute that it was requisite to
liquify the pus before their presence could be ascertained.
Fenwick also states that the progress is bad in propor-
tion to the amount of hepatic structure you may discover.
Where the ulcerative process has ceased, the particles
that have been thrown off lose their characteristic ap-
pearance. These are the cases that are most likely to re-
cover after tapping, and, therefore, it is important as re-
gards prognosis. In every instance the pus should be
carefully examined. He also thinks that the absence of
fragments of liver is a pretty sure indication that ulcera-
tion is not progressing. He gives several cases, which
seem to prove conclusively what he affirms; but as this
does not bear directly upon the points that I wish to
prove, I will refer the reader to the London Lancet of No-
vember 17th, 1877, where he will find all these cases re-
ported in full.
I did not give the history of any of these cases, but
simply the results of the examinations, as made and re-
ported by Dr. Fenwick.
Dr. Louis Starr, of Philadelphia, also reports a case of
liver abscess, in which a microscopic examination of the
pus revealed liver cells. See Transactions of College of
Physicians, Philadelphia, Third Series, Vol. II., page 17.
Conclusions.—I am confident, from the examinations
made by myself, and from the results of those reported by
the above authorities, that a careful microscopical exam-
ination of the pus from true hepatic abscesses ■will, in
every case, reveal liver cells, and with this fact proven
we certainly have an invaluable diagnostic point in cases
of hepatic abscesses.
				

